# Performance of ultra-sensitive malaria rapid diagnostic test to detect *Plasmodium falciparum* infection in pregnant women in Kinshasa, the Democratic Republic of the Congo

**DOI:** 10.1186/s12936-023-04749-2

**Published:** 2023-10-23

**Authors:** Japhet Kabalu Tshiongo, Flory Luzolo, Melissa Kabena, Lise Kuseke, Moussa Djimde, Patrick Mitashi, Crispin Lumbala, Kassoum Kayentao, Sandra Menting, Petra F. Mens, Henk D. F. H. Schallig, Pascal Lutumba, Halidou Tinto, Hypolite Muhindo Mavoko, Vivi Maketa

**Affiliations:** 1 Department of Tropical Medicine, University of Kinshasa (UNIKIN), Kinshasa, Democratic Republic of the Congo; 2 Amsterdam University Medical Centres, Department of Medical Microbiology and Infection Prevention, Laboratory for Experimental Parasitology, Academic Medical Centres at the University of Amsterdam, Amsterdam, The Netherlands; 3 Amsterdam Institute for Infection and Immunity, Infectious Diseases Programme, Amsterdam, The Netherlands; 4 Malaria Research and Training Center (MRTC), University of Sciences, Techniques and Technologies of Bamako (USTTB), Bamako, Mali; 5 Clinton Health Access Initiative, Kinshasa, Democratic Republic of the Congo; 6 Global Health Institute, Faculty of Medicine and Health Sciences, University of Antwerp, Antwerp, Belgium; 7 Institut Supérieur Des Techniques Médicales de Kinshasa (ISTM-Kinshasa), Kinshasa, Democratic Republic of the Congo; 8 Institut de Recherche en Sciences de La Santé - Clinical Research Unit of Nanoro (IRSS-CRUN), Nanoro, Burkina Faso

**Keywords:** Malaria, Pregnancy, Diagnosis, Rapid diagnostic test, Parasite density

## Abstract

**Background:**

Low peripheral parasitaemia caused by sequestration of *Plasmodium falciparum* in the placenta hampers the diagnosis of malaria in pregnant women, leading to microscopy or conventional rapid diagnostic tests (RDTs) false-negative results. Although mainly asymptomatic, maternal malaria remains harmful to pregnant women and their offspring in endemic settings and must be adequately diagnosed. Ultra-sensitive RDTs (uRDTs) are thought to be more sensitive than RDTs, and their diagnostic performance was assessed in the current study in pregnant women living in Kinshasa, a stable malaria transmission area in the Democratic Republic of the Congo.

**Methods:**

To assess and compare the diagnostic performances of both RDTs and uRDTs, 497 peripheral blood samples were tested using microscopy and quantitative polymerase chain reaction (qPCR) as the index and the reference tests, respectively. The agreement between the different diagnostic tests assessed was estimated by Cohen's Kappa test.

**Results:**

The median parasite density by qPCR was 292 p/μL of blood [IQR (49.7–1137)]. Using qPCR as the reference diagnostic test, the sensitivities of microscopy, RDT and uRDT were respectively [55.7% (95% CI 47.6–63.6)], [81.7% (95%CI 74.7–87.3)] and [88% (95% CI 81.9–92.6)]. The specificities of the tests were calculated at 98.5% (95% CI 96.6–99.5), 95.2% (95% CI 92.5–97.2) and 94.4% (95% CI 91.4–96.6) for microscopy, RDT and uRDT, respectively. The agreement between qPCR and uRDT was almost perfect (Kappa = 0.82). For parasite density (qPCR) below 100 p/µL, the sensitivity of RDT was 62% (95% CI 47.1–75.3) compared to 68% (95% CI 53.3–80.4) for uRDT. Between 100 and 200 p/µL, the sensitivity of RDT was higher, but still lower compared to uRDT: 89.4% (95% CI 66.8–98.7) for RDT versus 100% (95% CI 82.3–100) for uRDT. In both cases, microscopy was lower, with 20% (95% CI 10–33.7) and 47.3% (95% CI 24.4–71.1) respectively.

**Conclusions:**

uRDT has the potential to improve malaria management in pregnant women as it has been found to be slightly more sensitive than RDT in the detection of malaria in pregnant women but the difference was not significant. Microscopy has a more limited value for the diagnosis of malaria during the pregnancy, because of its lower sensitivity.

## Background

Malaria, defined as a peripheral or placental infection by *Plasmodium* parasites, is a major cause of morbidity and mortality among pregnant women and their offspring in Africa, including in the Democratic Republic of the Congo (DRC) [1–3]. Malaria in pregnancy is associated with several adverse maternal, fetal, and neonatal outcomes, such as maternal anaemia, miscarriage, preterm delivery and low birth weight [2]. Timely and accurate diagnosis is crucial to manage malaria and its complications. The prevalence of peripheral malaria in the DRC ranges from 24.2% to 69.9%, and that of placental malaria is 13.6% [4–6].

During pregnancy, *Plasmodium falciparum* parasites may be sequestered in the placenta, resulting in a false negative diagnostic test result in particular when using diagnostics with a relative low limit of detection, such as standard light microscopy on Giemsa-stained blood slides or conventional rapid diagnostic tests (RDTs) on peripheral blood [7, 8]. RDTs detect circulating *P. falciparum* antigens (most often histidine-rich protein 2, HRP2) and this should not be affected by the sequestration of parasites in the placenta. However, currently available RDTs are limited in their detection threshold and miss infections with low parasitaemia [9, 10]. The standard diagnostic method for detecting a malaria infection in DRC is microscopy and RDTs in the referral level but in the peripheral level like health centres, only RDTs are used [11].

Molecular techniques such as polymerase chain reaction (PCR) and loop mediated amplification (LAMP) can provide more sensitive diagnosis of malaria in pregnancy [12, 13]. Unfortunately, these molecular diagnostic technologies, in particular PCR, require sufficiently trained personnel, (sophisticated) equipment, reliable electricity and laboratory infrastructure, and are therefore difficult to implement in the routine diagnostic procedures for malaria in resource-limited settings. Another important bottleneck for the implementation of molecular methods is the nucleic acid extraction step, which is prone to errors and contamination.[14].

An innovative ultra-sensitive RDT (uRDT) (Alere™/Abbott Malaria Ag *P.F;* now called NxTek™ Eliminate Malaria *P. falciparum* RDT) has been developed [15, 16]. It uses an immunochromatographic membrane strip platform, similar to RDTs, to detect histidine-rich protein 2 (HRP2), has the same whole blood volume requirement (5 µL), a slightly longer time to results than the RDT but with improved analytical sensitivity with an advantage to detect low-density infections (40–125 pg/mL HRP2) and a claimed tenfold higher analytical sensitivity than that of RDTs [15, 17]. The uRDT also detected more than half of infections with a qPCR parasite density of 0.1–1.0 pRBC/μL in human blood samples from areas of high and low malaria transmission [15]. Review studies conducted in several malaria transmission settings have shown an improvement in the sensitivity and detection of malaria infection by the uRDT with PCR as the reference test. This gain varies from one malaria transmission context to another [18, 19]. In pregnant women, uRDT also showed different performance results between low and high malaria transmission area [20–22].

Considering that uRDT has a claimed better diagnostic performance than RDT [18], they may be suited to improve malaria diagnosis and screening in pregnant women [19]. It is relevant to assess the diagnostic performance of this test in our perennial high transmission setting in Kinshasa, because of the reported differences in performance of the uRDT from one setting to another, and the lack of data in DRC, where *P*. *falciparum* is the most important malaria species [1]. Furthermore, the RDT was reported not to be a perfect option for the intermittent screening and treatment (ISTp) strategy in pregnant women because of limited sensitivity [23]. Replacing RDT with more sensitive point-of-care test such as uRDT in ISTp would be an alternative but needs to be evaluated beforehand. Therefore, this study aimed to provide more evidence on the diagnostic performance of the uRDT compared with RDT using qPCR as gold standard for the diagnosis of *P. falciparum* malaria in peripheral blood of pregnant women in DRC.

## Methods

### Study design and population

This study was part of the ULTRAPYRAPREG project [24] (https://clinicaltrials.gov/study/NCT04783051). ULTRAPYRAPREG is a clinical trial that aims to assess whether intermittent screening with uRDT and treatment using pyronaridine-artesunate (IST-US-PA) is non inferior compared to the classic intermittent preventive treatment (using sulfadoxine-pyrimethamine, IPTp-SP) in terms of the proportion of maternal malaria, maternal anaemia, spontaneous abortions or intrauterine death during pregnancy, fetal morbidity and neonatal mortality at childbirth. Women were invited to participate in the study and after giving informed consent screened for malaria. Samples collected from these women were used in the present study as screening samples (n = 249). Next, women were randomized into one of the two study arms of the ULTRAPYRAPREG study. During the follow-up, women randomly selected to the ISTp-US-PA group were tested monthly from the beginning of the second semester of pregnancy with uRDT and treated with pyronaridine-artesunate (Pyramax®) when the test was positive. Women randomized to the IPTp-SP group received SP as recommended by the National Malaria Control Programme (NMCP) at weeks 16, 28, 32, and 36 of their pregnancy. A total of 250 pregnant women were enrolled at 2^nd^ trimester of pregnancy.

For the current study, available blood samples collected at screening (249 samples) and during active follow-up (248 samples, randomly selected from scheduled or unscheduled visits) were used. Follow-up samples were collected from one to 5 months after screening (baseline).

### Study site

This study was conducted at the Maternité Esengo, located in Kisenso, a semi-rural suburbs of Kinshasa, DRC. The facility has an average of 100 deliveries per month. Malaria transmission is perennial in DRC with around 4.3 million pregnancies at risk of *P*. *falciparum* infection and 1.35 million births affected by malaria infection yearly [25]. It is reported that 74.8% of pregnant women use insecticide-impregnated mosquito nets [26]. In addition, 73% and 60% of pregnant women have taken at least 2 or 3 doses of SP during the antenatal care visits [27]. Malaria prevalence in pregnant women is estimated at 39.7%, of which 95.3% of cases are due to *P. falciparum* [1, 6].

### Data and sample collection

Within the framework of ULTRAPYRAPREG, socio-demographic, clinical data, obstetric parameters and blood samples were collected at enrollment. Thereafter, each participant had scheduled monthly visits from enrollment to delivery and was advised to attend the study site at anytime in case of a health problem. At each visit, in addition to clinical and obstetric parameters, study staff performed a thick and thin blood smear, a RDT and a uRDT. In addition, blood spots were collected on filter paper (Whatman 3MM, 3 spots per card), which were dried thoroughly, put in individual zip-lock plastic bags containing silica-gel and stored at ambient temperature and shipped to the Netherlands for molecular analysis.

### Diagnostic test procedures

#### Index tests

##### RDTs and uRDTs

RDTs (SD Bioline® Malaria Ag *P. falciparum;* batches 05CDF061A-63A, 81A, 84A, 84A, 90A, 94A, Standard Diagnostics, Republic of Korea) and uRDTs (Alere Malaria Ag *P. falciparum* ultra-sensitive; batches 05LDF006B, 05LDG001B, 05BDDG043, 05LDG001A, Alere/Abbott, Republic of Korea, now called NxTek™ Eliminate Malaria *P. falciparum* RDT) were performed by trained technicians following the manufacturer’s instructions. Briefly, fresh capillary blood was used to perform the tests. Blood was applied to the sample port of the test followed by application of four drops of assay diluent. Twenty minutes (for uRDT) or fifteen minutes (for RDT) after application of the specimen, the result was interpreted. To ensure the validity of the RDT results, two independent laboratory technicians read the results within the set timeframe. In addition, a high-resolution photo of the test was taken and saved to serve for quality control. RDT was positive if the antigen and control lines were visible. The result was negative when only the control line was visible. When the control line was not visible, the RDT was invalid. In this case, it was repeated. In case of a discrepancy between two readers, a third reader’s opinion was sought.

##### Light microscopy

Thin and tick slides were prepared and stained with Giemsa 10% for 10 min. The thin smears were fixed with 100% methanol for 2 seconds before staining. The blood slides were examined by light microscopy at 1000 × magnification. The parasite density per microliter was determined on examining the thick slides and calculated using the following formula: Number of trophozoites × 8000/Number of white blood cells (WBC). All slides were independently read by two experienced microscopists who were blinded from the uRDT and RDT results. A slide was considered as negative when no parasite was identified after examining a minimum of 100 fields. In the case of discordant (results positive *versus* negative; parasitaemia difference > 20%; different species), a third reading was performed by an additional microscopist.

#### Reference test

##### qPCR

DNA was extracted from dried blood spots using the EMAG™ (BioMérieux, France), a platform for automatic nucleic acid extraction. Standard curves of *P. falciparum* 3D7 culture (10^4^–10^1^ parasites/μL) were used as positive controls and also negative controls (water) were included in every run in duplicate. Purified DNA was stored at—70 °C until further analysis. A 18S *P. falciparum* qPCRs was performed on a CFX96™ detection system (BioRad, Hercules, CA). The quantification was performed according to the standard curve. Results were analysed using CFX manager software (BioRad, Hercules, CA), with a set Baseline threshold of 100 relative fluorescence units. Samples were considered valid if they crossed this threshold within 40 PCR cycles. All samples with a value ≥ 1.0 parasite/µl were considered positive [28].

### Data management and statistical analysis

Study data were collected on CRF (case report form), and managed using REDCap electronic data capture tools hosted at University of Antwerp. Source data verification was performed by another operator to verify the information entered in the electronic CRF against the source document available on site. Analyses were done with Statistical Package for Social Sciences (SPSS) version 23. Sensitivity, specificity, positive and negative predictive values of uRDT (test index), RDT (test index) and microscopy (test index) and their 95% confidence intervals were calculated using the qPCR as a reference test with the MedCalc Software Ltd., diagnostic Test Evaluation Calculator, available at: https://www.medcalc.org/calc/diagnostic_test.php. The agreement between microscopy, RDT, uRDT and the qPCR was assessed on all samples by determination of Cohen's Kappa coefficient and interpreted according to the scoring system described by Landis and Koch [29]. McNemar’s test was used to determine significant difference between the diagnostic performance of the test. *P*-values of < 0.05 were considered significant.

## Results

### Demographic and obstetrical characteristics of pregnant women

**Fig. 1:**
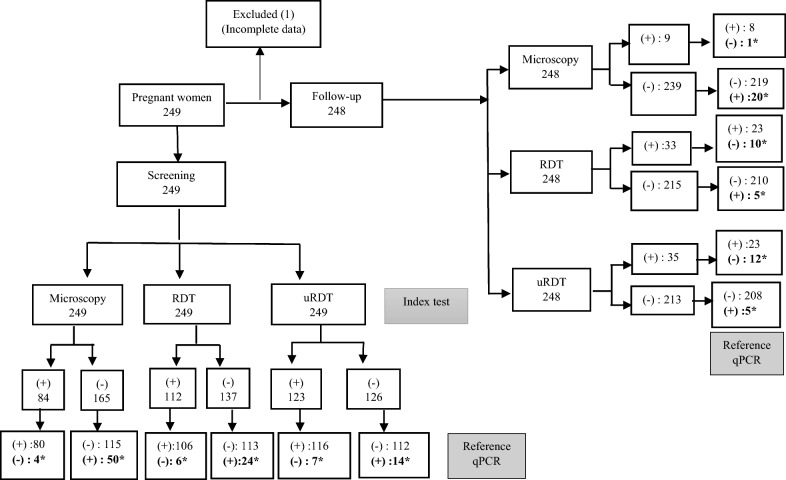
Flow of pregnant women and results of tests used. The flow chart shows the number of pregnant women during screening and follow-up, the number of blood samples collected and the results of each test used. * Discrepancy observed when compared to the reference test; (+): positive; (-): Negative; RDT (rapid diagnostic test); uRDT (ultra-sensitive rapid diagnostic test); qPCR (quantitative polymerase chain reaction)

Overall, 249 samples were collected at inclusion (baseline) and 248 samples were selected from follow-up visits (Fig. 1). The demographic and obstetrical characteristics are presented in Table 1. The age ranged from 18 to 42 years with a median of 25 years [(IQR (21–31) and the median gestational age was 22.5 weeks (range 16–41). According to gravidity, 37.7% (94/249), 20.8% (52/249), and 41.3% (103/249) were primi, secondi, and multigravidae, respectively. Fifty percent (50.1%) of blood samples were collected in the second trimester of pregnancy (screening) and 49.8% in the third trimester (follow-up visit).

**Table 1:** Baseline characteristics of pregnant women enrolled and followed-up in the study

	All samples (N = 497)	Screening samples (N = 249)	Follow-up samples (N = 248)
Age (years): median (IQR)	25 (21–31)	25 (21–31)	25 (21–31)
Gestational age (weeks): median (IQR)	22.5 (18–34)	18 (17–20)	34 (32–37)
Gravidity: median (IQR)	2 (1–4)	2 (1–4)	–
Primigravida: (n (%))	–	94 (37.8%)	–
Secundigravida: (n (%))	–	52 (20.9%)	–
Multigravida: (n (%))	–	103 (41.4%)	–
Microscopy positive (n (%))	93 (18.7%)	84 (33.7%)	9 (3.6%)
Microscopy PD (p/µl): median (IQR)	2160 (740–5080)	1960 (730–5010)	4480 (1341–8800)
< 200 (n (%))	6 (6.5%)	5 (6.0%)	1 (11.1%)
200–2000 (n (%))	39 (41.9%)	38 (45.2%)	1 (11.1%)
> 2000 (n (%))	48 (51.6%)	41(48.8%)	7 (77.8%)
qPCR positive (n (%))	158	130 (52.2%)	28 (11.2%)
qPCR PD (p/µl): median (IQR)	292 (49.7–1137)	334 (66–1230)	99 (10–858)
< 200 (n (%))	69 (43.7%)	53 (40.8%)	16 (57.1%)
200- 2000 (n (%))	68 (43.0%)	58 (44.6%)	10 (35.7%)
> 2000 (n (%))	21 (13.3%)	19 (14.6%)	2 (7.1%)

IQR (Interquartile); N (sample size); PD: parasite density; p/µl: parasite per microliter

### Malaria infection rate as determined by different diagnostic techniques

Malaria infection was detected by uRDT in 31.8% (158/497) (95% CI 27.7–36.1), either during enrolment and/or follow-up (Table 2). This rate was similar to qPCR 31.8% (158/497) (95% CI 27.7–36.1), but higher compared to RDT 29.2% (145/497) (95% CI 25.2–33.4). In contrast, microscopy was positive for only 18.7% (93/497) (95% CI 15.4–22.4) of the cases. The differences between uRDT detection, RDT detection and microscopy were statistically significant (p < 0.001). The median parasite density by microscopy was 2,160 parasites per μL of blood [(IQR (740.0–5,080.0) parasites per μL of blood (range 23–25,040)]. The median parasite density was 292 parasites per μL of blood [(IQR (49.7–1137) parasites per μL of blood (range 1–13,300)] among qPCR positive participants. Among pregnant women with malaria as confirmed by microscopy, 51.6% had a parasite density above 2,000 parasites per μL of blood. During the enrolment, the malaria infection was the highest [52.2% (130/249] (95% CI 45.8–58.6) by qPCR. uRDT gave a positive result in 49.4% (123/249) (95% CI 43.0–55.8) of the participants, RDT in 45.0% of the participants (112/249) (95% CI 38.7–51.4) and microscopy in 33.7% of the participants (84/249)(95% CI 27.9–40.0). Among microscopy positive participants at enrolment, the median parasite density was 1,960 p/μL of blood [(IQR 730–5010 p/μL of blood (range 23–25,040)]. During the follow-up, the uRDT [35/248 (14.1%) (95% CI 10.0–19.1)], RDT [33/248 (13.3%) (95% CI 9.3–18.2)] and qPCR 28/248 (11.3%) (95% CI 7.6–15.9) performed equally but detected significantly more malaria infection than microscopy [9/248(3.6%) (95% CI 1.7–6.8)] (p < 0.001).

**Table 2:** Positivity rate detected by the different diagnostics at the screening and during follow-up

Test	All samples (N = 497)	Screening samples (N = 249)	Followup samples (N = 248)
Microscopy positive	93 (18.7%)	84 (33.7%)	9 (3.6%)
RDT positive	145 (29.2%)	112 (45.0%)	33 (13.3%)
uRDT positive	158 (31.8%)	123 (49.4%)	35 (14.1%)
qPCR positive	158 (31.8%)	130 (52.2%)	28 (11.3%)

N (sample size); RDT (rapid diagnostic test), uRDT (ultrasensitive rapid diagnostic test); qPCR(quantitative polymerase chain reaction)

### Performance of the different diagnostic tests

First, the diagnostic performance of all employed tests on all samples collected in this study was analysed. Using qPCR as the reference test, uRDT had an overall higher sensitivity [88.0% (95% CI 81.9–92.6)], when compared to microscopy [55.7% (95% CI 47.6–63.6)] (p < 0.001), and a comparable sensitivity to RDT [81.7% (95% CI 74.7–87.3)] (Table 3). Thus, the uRDT detected more true positive pregnant women than microscopy and RDT, but also gave more false positives results. Microscopy specificity tended to be the highest [98.5% (95% CI 96.6–99.5)] but the difference was not significant (p = 0.059). Microscopy specificity was followed by the RDT [95.2% (95% CI 92.5–97.2)] which was similar to the specificity of uRDT [94.4% (95% CI 91.4–96.6)]. The positive predictive values (PPV) and negative values (NPV) of uRDT and RDT were comparable. The agreement between uRDT and qPCR was almost perfect, Kappa = 0.82 (95%CI 0.77–0.87).

**Table 3:** Overall diagnostic accuracy of microscopy, RDT, and uRDT compared to qPCR as a reference test for the diagnosis of malaria

		qPCR		Total	Sensitivity (95% CI)	Specificity (95% CI)	Value (95%CI)				
		(+)	(-)				PPV (95% CI)	NPV (95% CI)	LR(+) (95% CI)	LR(-) (95% CI)	Kappa (95% CI)
Microscopy	(+)	88	5	93	55.7% (47.6–63.6)	98.5% (96.6–99.5)	94.6% (88.0–97.7)	82.7% (80.0–85.0)	37.8 (15.7–91.1)	0.46 (0.4–0.5)	0.60 (0.53–0.68)
	(-)	70	334	404							
RDT	(+)	129	16	145	81.7% (74.7–87.3)	95.2% (92.5–97.2)	89% (83.2–92.9)	91.7% (88.9 – 94.0)	17.3 (10.7–28.1)	0.19 (0.1–0.3)	0.78 (0.72–0.84)
	(-)	29	323	352							
uRDT	(+)	139	19	158	88% (81.9–92.6)	94.4% (91.4–96.6)	88% (82.5–91.9)	94.4% (91.7–96.2)	15.7 (10.0–24.4)	0.13 (0.1–0.2)	0.82 (0.77–0.87)
	(-)	19	320	339							

Pf (P. *falciparum*); RDT (rapid diagnostic test); uRDT (ultrasensitive rapid diagnostic test); qPCR (quantitative polymerase chain reaction); (+) (positive); (-) (negative); PPV (positive predictive value); NPV (negative predictive value); CI (confidence interval);LR: Likelihood ratio

In Table 4, an analysis is presented on the performance of the different diagnostic tests when either screening samples or follow-up samples were used. The sensitivity of the uRDT [89.2% (95% CI 82.6–94)] and RDT [81.5% (95% CI 73.8–87.8)] was higher compared to microscopy 61.5% (95% CI 52.6–69.9) on samples that were tested on enrolment (Table 4). The specificity of the three tests was similar. During the follow-up, the sensitivity of uRDT and RDT was similar. The sensitivity of microscopy [28.6% (95% CI 13.0–48.0)] was low. The specificity of microscopy was very good 99.6% (95% CI 97.5–100) when tested on follow-up samples and almost similar to the specificity of both RDT (95.5%; 95% CI 91.8–97.8) and uRDT (94.6%; 95% CI 90.7–97.1).

**Table 4:** Comparison of microscopy, RDT, uRDT for detecting *P. falciparum* during the screening and the follow-up

	Test		qPCR		Value (95% CI)				LR(+)	LR(-)	Kappa (95%CI)
					Sensitivity	Specificity	PPV	NPV			
Screening (N = 249)	Microscopy		(+)	(-)							
		(+)	80	4	61.5% (52.6–69.9)	96.6% (91.6–99.0)	95.2 (88.3–98.1)	69.7 (64.9–74.1)	18.3 (6.9–48.4)	0.4 (0.32–0.5)	0.57 (0.48–0.66)
		(-)	50	115							
	RDT	(+)	106	6	81.5% (73.8–87.8)	95.0% (89.4–98.1)	94.6% ( 89–97.5)	82.5% (76.6–87.1)	16.2 (7.4–35.4)	0.2 (0.1–0.3)	0.76 (0.68–0.84)
		(-)	24	113							
	uRDT	(+)	116	7	89.2% (82.6–94)	94.1% (88.2–97.6)	94.3% (89–97.1)	88.9 (83–92.9)	15.2 (7.4–31.2)	0.1 (0.1–0.2)	0.83 (0.76–0.9)
		(-)	14	112							
Follow-up (N = 248)	Microscopy	(+)	8	1	28.6% (13.2–48.7)	99.6% (97.5–100)	88.9% (51–98.4)	91.6% (89.7–93.2)	62.9 (8.2–484.1)	0.72 (0.57–0.91)	0.4 (0.2–0.6)
		(-)	20	219							
	RDT	(+)	23	10	82.1% (63.1–93.9)	95.5% (91.8–97.8)	69.7% (55.0–81.2)	97.7 (94.0–98.7)	18.1 (9.6–33.9)	0.19 (0.1–0.4)	0.72 (0.7–0.85)
		(-)	5	210							
	uRDT	(+)	23	12	82.1% (63.1–94)	94.6% (90.7–97.1)	65.7% (51.9–77.3)	97.7% (95–98.9)	15.1 (8.5–26.8)	0.2 (0.1–0.4)	0.69 (0.55–0.83)
		(-)	5	208							

RDT (rapid diagnostic test); uRDT (ultra-sensitive rapid diagnostic test); qPCR (quantitative polymerase chain reaction); ( +) (positive); (-) (negative); PPV (positive predictive value); NPV (negative predictive value); CI (confidence interval); N (sample size); LR( +):likelihood ratio

The agreement between uRDT and qPCR was almost perfect Kappa = 0.81 (95% CI 0.76–0.9) during the screening and substantial Kappa = 0.69 (95% CI 0.55–0.83) during the follow-up. The agreement between RDT and qPCR showed a substantial agreement both during the screening (Kappa = 0.76) and follow-up (Kappa = 0.72). There is no significant difference between the performance of the RDT and uRDT (p = 0.061). However the uRDT seems to perform slightly better compared to RDT during screening and RDT seems to perform slightly better during follow-up when qPCR is considered as reference test (see Table 4).

Table 5 presents the results of performance of diagnostic by gravidity. The sensitivity of uRDT is higher compared to RDT in gravidae. This was followed by the sensitivity of RDT 91.3% (95% CI 81.0–97.1) in primigravidae and in the secundigravidae 81.4% (95% CI 61.9–93.7%). The sensitivity of microscopy, RDT and uRDT was lowest in the multigravidae for all three tests (Table 5). The agreement between microscopy and qPCR is moderate in blood samples collected to primigravidae and multigravidae and substantial in segundigravidae. The agreement between uRDT and RDT and qPCR was almost perfect in primigravidae and almost perfect to substantial in secundigravidae.

**Table 5:** Comparison of microscopy, RDT, uRDT for detecting *P. falciparum* in Primi, Secundi and multigravidae at baseline

			qPCR		Value (95%CI)				LR (+) (95%CI)	LR (-) (95%CI)	Kappa (95%CI)
					Sensitivity (95%CI)	Specificity (95%CI)	PPV (95%CI)	NPV (95%CI)			
Primigravidae (N = 94)	Test		(+)	(-)							
	Microscopy	(+)	39	2	67.2% (53.6–78.9)	94.4% (81.3–99.3)	95.1 (83.3–98.7)	64.1 (55.1–72.2)	12.1 (3.1–47.1)	0.3 (0.2–0.5)	0.57 (0.41–0.71)
		(-)	19	34							
	RDT	(+)	53	3	91.3% (81–97.1)	91.7% (77.5–98.2)	94.6% ( 85.6–98.1)	86.8% (74–93.9)	11.0 (3.7–32.5)	0.09 (0.0–0.2)	0.82 (0.7–0.93)
		(-)	5	33							
	uRDT	(+)	56	4	96.6% (88–99.6)	88.9% (73.9–96.9)	93.3% (84.7–97.3)	94.1 (80.3–98.4)	8.7 (3.4–21.9)	0.0 (0.0–0.1)	0.86 (0.76–0.96)
		(-)	2	32							
Secundigravidae (N = 52)	Microscopy	(+)	17	0	63% (42.3–80.6)	100% (86.2–100)	100%	71.4% (60.4–80.4)	-	0.37 (0.2–0.6)	0.62 (0.42–0.81)
		(-)	10	25							
	RDT	(+)	22	1	81.5% (61.9–93.7)	96% (79.7–99.9)	95.7% (76.1–99.3)	82.8 (68.4–91.4)	20.4 (3.0–140.2)	0.2 (0.1–0.4)	0.77 (0.6–0.94)
		(-)	5	24							
	uRDT	(+)	26	1	96.3% (81–99.9)	96% (79.7–99.9)	96.3% (79.1–99.4)	96.6% (77.8–99.4)	24.1 (3.5–164.5)	0.0 (0.0–0.3)	0.92 (0.81–1)
		(-)	1	24							
Multigravidae (N = 103)	Microscopy	(+)	24	2	53.3% (36.9–68.3)	96.6% (88.0 – 99.6)	92.3% (75–98)	72.7% (66–78.5)	15.5 (3.9–62.0)	0.5 (0.3–0.7)	0.52 (0.4–0.7)
		(-)	21	56							
	RDT	(+)	31	2	68.8% (53.3–81.8)	96.6% (88.0–99.6)	93.9% (79.7–98.4)	80% (72.0–86.1)	20.0 (5.0–79.0)	0.3 (0.2–0.5)	0.67 (0.53–0.81)
		(-)	14	56							
	uRDT	(+)	34	2	75.6 (60.5–87.1)	96.5% (88.0–99.6)	94.4% (81.2–98.5)	83.5% (75.2–89.5)	22.0 (5.6–86.4)	0.2 (0.1–0.4)	0.73 (0.6–0.87)
		(-)	11	56							

RDT (rapid diagnostic test); uRDT (ultra-sensitive rapid diagnostic test); qPCR (quantitative polymerase chain reaction); ( +) (positive); (-) (negative); PPV (positive predictive value); NPV (negative predictive value); CI (confidence interval); N (sample size); LR: Likelihood ratio

In Table 6, the sensitivity of the different tests was assessed when the samples were stratified according to parasite density by qPCR. Low density parasitaemia (< 100 p/µL of blood by qPCR) [30] infections were observed in 10% (50/497) of the participants. In this group uRDT had a sensitivity of 68% (95% CI 53.3–80.4), co- RDT, 62% (95% CI 47.1–75.3) and microscopy 20% (95% CI 10.0–33.7). In the group of 100–200 p/ µl of blood all the tests had an increased sensitivity with uRDT detecting all positive samples (100% (95% CI 82.3–100), RDT 89% and microscopy just below 50% of the samples. At parasite density > 200 parasites, uRDTs missed three samples that were found positive by PCR and this resulted in a sensitivity of 96.6% (95% CI 90.4–99.3). RDT had a sensitivity of 91% (95% CI 83.0–96.0) and microscopy reported the lowest sensitivity. In low density blood samples, the agreement between uRDT and RDT was almost perfect (Kappa = 0.86).

**Table 6:** Comparison of microscopy, uRDT, and RDT sensitivity stratified by parasite density (p/μL) as determined by qPCR

Parasite density	qPCR (+)	Microscopy (+)	Microscopy sensitivity (95% CI)	RDT(+)	RDT sensitivity (95% CI)	uRDT (+)	uRDT Sensitivity (95% CI)
< 100	50	10	20% (10.0–33.7)	31	62% (47.1–75.3)	34	68% (53.3–80.4)
100–200	19	9	47.3% (24.4–71.1)	17	89.4% (66.8–98.7)	19	100% (82.3–100)
> 200	89	69	77.5% (67.4–85.7)	81	91% (83.0–96.0)	86	96.6% (90.0–99.3)

RDT (rapid diagnostic test); uRDT (ultra-sensitive rapid diagnostic test); qPCR (quantitative polymerase chain reaction); (+) (positive); (-) (negative); PPV (positive predictive value); NPV (negative predictive value); CI (confidence interval); N (sample size)

## Discussion

This study has evaluated uRDT for malaria under field conditions in pregnant women in DRC, an area of high malaria transmission. Previous studies evaluating uRDTs have determined their accuracy under laboratory conditions [17, 21, 22], or in populations living in settings with low to moderate high malaria transmission [31, 32].

The present study showed that of the use of uRDT to detect a malaria infection during pregnancy can be an improvement in terms of sensitivity when this is compared to RDT and microscopy. This improvement in sensitivity is especially true in low parasite density infections before initiation of malaria treatment. The uRDT was the test with the highest sensitivity in primi, secondi and multi gravidae compared to other malaria diagnostics employed in the present study.

For all samples, uRDT detected malaria at a similar prevalence as qPCR which is higher than the detection of RDT and microscopy. In addition, uRDT and qPCR detected more malaria infections than microscopy during follow-up visits. This observation highlighted the likely underestimation of malaria infections by the tests routinely used in pregnant women with low-density parasitemia levels as observed during the follow-up period.

Using qPCR as the reference diagnostic test, uRDT showed overall a higher sensitivity compared to microscopy and comparable to RDT. This is in line with a study conducted in pregnant women in Benin where it was reported that uRDT had higher sensitivity (60.5%) compared to RDT at 44.2% at a mean parasite density of 20.7 p/μL [20]. The sensitivity rates in the DRC setting are higher than those observed in the Benin study. This is probably due to the high median parasite density by qPCR of 292: p/uL of blood which may result in high concentration of HRP2, detected by uRDT [19]. In addition, the sensitivity of uRDT in this study remained higher than RDT at a parasite density > 200 p/uL of blood. The results observed in Indonesia contrast with this study, the sensitivity of uRDT and RDT remained very low [19.6%(95% CI 13.9–26.8) vs 22.8% (95% CI 16.7–30.3)] [32]. Furthermore, Acquah et al. have showed that RDT and uRDT have the same sensitivity of 53.8% in the subpopulation of pregnant women in Ghana [33]. In this study, the sensitivity of uRDT decreases with increasing gravidity. These results are similar to those found in Kenya, where uRDT detected more (59.4%) malaria infection than RDT in febrile primary and secondary gravidae women and decreased to 50% in febrile multigravida women, although at a lower level than DRC study [34].

Aquach et *al*. reported a sensitivity of 52.4% for uRDT and 42.9% for RDT in the afebrile non-pregnant population in Ghana [33]. Nevertheless, the sensitivity in the DRC was higher than in Ghana. The gain in sensitivity can be explained by two reasons. Firstly, Kinshasa has a high malaria transmission and consequently pregnant women are potentially exposed to multiple infectious bites per week [35], resulting in relatively high parasite densities as observed in our results (Table 1). Secondly, the placenta of pregnant women serves as an extra site for harboring parasites [36] and thus allowing the release of additional HRP2 antigen, which is the diagnostic target of the uRDT, from the parasites sequestered within the placenta [33].

Using qPCR as a reference for true positive and negative cases, the sensitivity of uRDT was higher than RDT and microscopy during enrollment. In addition, this sensitivity was higher at enrollment than at follow-up. This result was similar to that found by Turnbull et al. in children in Kenya where the sensitivity of uRDT was higher than microscopy [37].

Although more sensitive than RDT, the uRDT is less specific than RDT in the present study. This was also observed in a another study in non-pregnant individuals [33]. The lower specificity of uRDT could be attributed to the ability of this test to detect low concentrations of HRP2 persisting after the elimination of parasites from the blood thus resulting in false positive results [38]. This same phenomenon of producing more false positive results than RDT for malaria infection detection in pregnant women was observed in the present study. Therefore the uRDT cannot be used as a tool to determine therapeutic success in pregnant women. Overall, the level of agreement between qPCR and uRDT was almost perfect and slightly higher to RDT.

The advantage of uRDT over the other diagnostic options seems especially relevant at low parasite densities. In blood samples with low parasitaemia (< 100 p/µL blood), uRDT detected more malaria infections in the present study than RDT and microscopy. Missing low density infections in pregnant women and especially associated with lack of symptoms, contribute to onward malaria transmission in the community but also may have adverse consequences on the mother and fetus. Improving malaria screening followed by an effective treatment of positive cases can reduce the silent reservoir infections. Although uRDT still missed almost one third of low density infections and thus seemed not to be the ultimate answer for low density infections in pregnant women, this test outperformed the other tests that were evaluated in this study.

A limitation of the current study is the fact that samples that were positive in qPCR and negative in RDT or uRDT were not tested on the presence of the *Pf* HRP2 gene. *pf*HRP2 is a gene that codes for HRP2, the target antigen in these RDTs. Recent studies have shown that HRP2 gene deletions are leading to test failures and that these deletions are spreading [39, 40]. However, *pf*HRP2 gene deletions have not been detected in Kinshasa [41], but a proportion of 0.27% was reported in others districts of DRC [42]. In future studies on HRP2 based RDTs the prevalence of HRP2 deletions should be assessed. Not only to interpret the results itself but also to asses if the test is suitable for the area it is intended for. The strength of the current study is field conditions, stratification of densities, and gravidity and the follow-up evaluation.

This has been the first study investigating uRDT in pregnant women under field conditions. This study showed that uRDT is a sensitive method in detecting malaria in pregnancy. This test could be used at every antenatal care visit for malaria screening [43]. This is even more true for areas where malaria transmission is intense. However, at the moment uRDTs are not available on the market in DRC and their cost-effectiveness needs to be assessed in comparison to RDTs. The current implementation seems to be especially hampered by its high cost, long delivery time after ordering and availability compared to RDT. These issues need to be addressed before implementation. The cost-effectiveness of integrating this new tool into maternal health programme in high-transmission areas as DRC needs to be evaluated.

## Conclusion

In conclusion, the uRDT was slightly more sensitive than RDT, but far better than microscopy, especially during the screening and in primigravidae, for the detection of a malaria infection. In malaria-endemic areas, the uRDT can provide added value by detecting more malaria infections than the RDT and the microscopy. In addition uRDT detects more cases in low density carriers *P. falciparum*. This indicates the potential value of uRDT in the management of malaria during pregnancy.

## Data Availability

Data are available from the corresponding author on reasonable request.

## Abbreviations

CI: Confidence interval
RDT: Rapid diagnostic test
DRC: Democratic Republic of the Congo
DNA: Deoxyribonucleic acid
HRP2: Histidine-rich protein 2
IQR: Inter quartile
LAMP: Loop mediated amplification
N: Sample size
NPV: Negative predictive value
PD: Parasite density
pg/mL: Picogram per millilitre
p/µL: Parasite per microlitre
pRBC: Parasitized red blood cell
PPV: Positive predictive value
qRT-PCR: Quantitative real time polymerase chain reaction
qPCR: Quantitative polymerase chain reaction
uRDT: Ultra-sensitive rapid diagnostic test
WBC: White blood cell
